# Beyond Disease Activity: Fatigue and Health-Related Quality of Life in Rheumatoid Arthritis Remission and Low Disease Activity

**DOI:** 10.3390/jcm15187133

**Published:** 2026-09-14

**Authors:** Joan M. Nolla, Lidia Valencia-Muntalà, Diego Benavent, Paola Vidal-Montal, Laura Berbel-Arcobé, Montserrat Roig-Kim, Martí Aguilar-Coll, Mònica Cubells, Aina Fabregat, Javier Narváez, Carmen Gómez-Vaquero

**Affiliations:** Department of Rheumatology, IDIBELL-Hospital Universitari de Bellvitge, University of Barcelona, 08035 Barcelona, Spain

**Keywords:** rheumatoid arthritis, disease activity, remission, low disease activity, DAS28, RAPID3, fatigue, health-related quality of life, cross-sectional study

## Abstract

**Background/Objectives:** Remission and low disease activity (LDA) are established treatment targets in rheumatoid arthritis (RA), but they may not imply normalization of patient-perceived disease burden. Fatigue and health-related quality of life (HRQoL) provide complementary measures of patient-perceived health beyond inflammatory disease activity. Moreover, disease activity states defined by the Disease Activity Score in 28 joints (DAS28) and the Routine Assessment of Patient Index Data 3 (RAPID3) may be associated with different patient-reported outcome profiles. We aimed to compare fatigue and HRQoL in patients with RA classified as being in remission or LDA with those of controls and to assess agreement between DAS28 and RAPID3 classifications. **Methods:** We conducted a cross-sectional comparative study with a control group within the BELL-RA-LIFE cohort including 275 patients with RA aged >50 years and 300 controls with a comparable age and sex distribution. Fatigue was assessed using the Functional Assessment of Chronic Illness Therapy-Fatigue (FACIT-F) and HRQoL using the Short Form-12 Physical (PCS) and Mental Component Summary (MCS) scores. Patients were classified independently according to DAS28 and RAPID3. Multivariable linear regression models adjusted for age, sex, body mass index, and hemoglobin were used to estimate differences versus controls. Agreement between DAS28 and RAPID3 was assessed using weighted Cohen’s kappa. **Results:** No statistically significant differences from controls were detected in FACIT-F or MCS scores among patients in DAS28 remission, although PCS scores were lower (adjusted difference −3.37, 95% CI −5.56 to −1.18; nominal *p* = 0.003; Holm-adjusted *p* = 0.033). DAS28-defined LDA was associated with greater fatigue and poorer PCS scores, although these differences did not remain statistically significant after Holm adjustment (adjusted *p* = 0.256 and *p* = 0.100, respectively), whereas MCS scores remained significantly lower (adjusted difference −5.15, 95% CI −8.14 to −2.15; nominal *p* = 0.001; adjusted *p* = 0.012). In patients in RAPID3 remission, PCS and MCS scores did not differ significantly from controls, while FACIT-F scores were higher; this difference did not remain statistically significant after Holm adjustment (adjusted *p* = 0.117); no statistically significant differences were detected for RAPID3-defined LDA across the three outcomes. Overall agreement between DAS28 and RAPID3 was limited (quadratic weighted κ = 0.35, 95% CI 0.26–0.44), with no significant difference between women and men (κ = 0.30 and 0.41, respectively; Δκ = 0.11, 95% CI −0.12 to 0.31; *p* = 0.34). RAPID3 more frequently classified patients into higher disease activity categories than DAS28 in 80.6% of discordant classifications. **Conclusions:** Remission and LDA in RA were associated with different residual health burdens when assessed using DAS28. Patients in DAS28 remission had significantly lower physical HRQoL than controls, while those in DAS28-defined LDA had significantly poorer mental HRQoL. No statistically significant differences in fatigue or HRQoL were observed for RAPID3-defined remission or LDA. Agreement between DAS28 and RAPID3 was limited and directional, with RAPID3 more frequently assigning patients to higher disease activity categories. These findings support the complementary assessment of inflammatory disease activity and patient-reported health in patients with RA.

## 1. Introduction

The implementation of treat-to-target strategies has established remission and low disease activity (LDA) as the principal therapeutic goals in rheumatoid arthritis (RA) [1,2]. Composite disease activity indices are central to this approach and are routinely used to guide treatment decisions [1,2]. However, achieving a predefined disease activity state, as determined by commonly used assessment instruments, does not necessarily imply complete resolution of the impact of RA from the patient’s perspective [3].

Persistent fatigue, impaired physical function and reduced health-related quality of life (HRQoL) have been reported even among patients who fulfil conventional criteria for remission [3,4]. This discrepancy has contributed to the concept of residual disease burden, whereby clinically controlled inflammatory disease may coexist with symptoms and functional consequences that remain relevant to patients. Fatigue and HRQoL are particularly informative in this setting because they capture complementary dimensions of disease impact beyond inflammatory activity [4,5]. Fatigue reflects the symptomatic burden experienced by the patient, whereas HRQoL integrates the broader physical and mental consequences of disease. Together, they provide a clinically meaningful framework for assessing whether remission or LDA is accompanied by recovery towards the health status of individuals without RA.

Importantly, remission and LDA are commonly considered acceptable treatment targets in clinical practice, but they may not represent equivalent states in terms of fatigue and HRQoL [1,2,6,7]. Most studies addressing residual disease burden compare outcomes across disease activity categories within RA populations [3,6,7,8]. Although clinically informative, this approach does not establish how fatigue and HRQoL in patients who are in remission or LDA differ from those observed in individuals without RA. The inclusion of a control group therefore provides a relevant external reference against which the extent of residual impairment can be more meaningfully interpreted.

The interpretation of disease control may also depend on the instrument used to define it. The Disease Activity Score in 28 joints (DAS28) combines tender and swollen joint counts obtained by clinical examination, an acute-phase reactant reflecting systemic inflammation, and patient global assessment [9]. In contrast, the Routine Assessment of Patient Index Data 3 (RAPID3) is based entirely on patient-reported measures of physical function, pain and global health [10]. Consequently, DAS28 and RAPID3 may capture partly different dimensions of RA and may classify the same patient into different disease activity states [10,11,12]. Whether remission or LDA defined by these instruments has the same clinical meaning in terms of fatigue and HRQoL remains uncertain.

In the present study, conducted within the BELL-RA-LIFE cohort, we sought to compare fatigue and HRQoL outcomes in patients classified as being in remission or LDA with those of individuals without RA, according to DAS28- and RAPID3-defined disease activity states. As a secondary objective, we assessed the agreement between DAS28 and RAPID3 classifications and the direction of discordance between both instruments.

## 2. Materials and Methods

### 2.1. Study Design and Population

We conducted this observational cross-sectional comparative study with a control group within the BELL-RA-LIFE cohort at the Department of Rheumatology of Hospital Universitari de Bellvitge, Barcelona, Spain.

BELL-RA-LIFE is a single-center clinical cohort designed to characterize the overall impact of RA across aging in routine clinical practice. Its assessment framework includes comorbidity, nutritional and immune-related status, physical function, patient-reported outcomes, and HRQoL. Previous analyses derived from BELL-RA-LIFE have addressed specific dimensions of this multidimensional disease burden, including fatigue, sarcopenia and osteosarcopenia, nutritional risk, and HRQoL, using either the complete cohort or age- or sex-defined subsets [13,14,15,16,17,18,19]. Consequently, participant populations and some clinical and patient-reported variables overlap across publications. Importantly, the present analysis addresses a distinct question that has not previously been examined in this cohort: whether remission and low disease activity defined by DAS28 or RAPID3 are associated with differences in fatigue and physical and mental HRQoL relative to controls, together with the agreement and direction of discordance between the two disease activity classifications.

We consecutively enrolled patients during routine rheumatology follow-up between June 2020 and December 2023. Eligibility required an age ≥ 50 years and fulfillment of the 2010 ACR/EULAR classification criteria for RA. The study population comprised 275 patients with RA, including 191 women (69.5%) and 84 men (30.5%).

### 2.2. Control Group

The control group included 300 participants without inflammatory arthritis, recruited from the same geographical and healthcare setting as the RA cohort and selected to provide a comparable age and sex distribution. We recruited controls from several sources, including relatives accompanying patients to outpatient visits, individuals assessed for non-inflammatory musculoskeletal complaints, predominantly soft-tissue disorders, and persons attending the hospital for conditions unrelated to the musculoskeletal system.

We applied the same exclusion criteria to both groups. We excluded individuals with active cancer or advanced cardiac, respiratory, hepatic, or renal disease to minimize the potential influence of major systemic conditions on the outcomes evaluated.

### 2.3. Demographic, Clinical and Laboratory Assessment

We recorded age, sex, and body mass index (BMI) in both patients with RA and controls. BMI was calculated as body weight in kilograms divided by height in meters squared.

For patients with RA, we collected clinical information during this study visit and supplemented it with data from the medical record. Disease-related variables included disease duration, rheumatoid factor and anti-citrullinated peptide antibody status, and current treatment. We classified pharmacological therapy as glucocorticoids, conventional synthetic disease-modifying antirheumatic drugs (csDMARDs), biological disease-modifying antirheumatic drugs (bDMARDs), or Janus kinase inhibitors.

We measured hemoglobin in both study groups. In patients with RA, we also obtained erythrocyte sedimentation rate (ESR) and C-reactive protein (CRP) from the laboratory assessment closest to this study visit.

### 2.4. Disease Activity Assessment

We evaluated inflammatory disease activity using the erythrocyte sedimentation rate-based Disease Activity Score in 28 joints (DAS28-ESR), hereafter referred to as DAS28 throughout the manuscript. DAS28 incorporates tender and swollen joint counts, patient global assessment, and ESR. We categorized patients as being in remission (DAS28 < 2.6), LDA (2.6–3.2), moderate disease activity (>3.2–5.1), or high disease activity (>5.1) [9].

We also evaluated disease status using the RAPID3, which combines patient-reported physical function, pain, and global assessment. RAPID3 categories were remission (≤3), LDA (3.01–6), moderate disease activity (6.01–12), and high disease activity (>12), according to previously established RAPID3 disease activity categories [10].

We generated DAS28 and RAPID3 classifications independently for each patient. For the present analyses, comparisons of fatigue and HRQoL with controls focused on patients classified as being in remission or LDA according to each instrument.

### 2.5. Fatigue Assessment

We measured fatigue in both patients with RA and controls using the Functional Assessment of Chronic Illness Therapy-Fatigue (FACIT-F) questionnaire [20]. We treated FACIT-F scores as a continuous variable, with higher values indicating less fatigue. We also used a threshold of <40 to identify clinically relevant fatigue [21].

### 2.6. Health-Related Quality of Life

Health-related quality of life was assessed using the Spanish-language SF-12 version 1. Physical Component Summary (PCS) and Mental Component Summary (MCS) scores were calculated according to the standard SF-12v1 scoring algorithm based on weighted item coefficients [22], with higher scores indicating better health-related quality of life. In the present study, scores were interpreted by comparison with the control group rather than against an external normative population.

### 2.7. Statistical Analysis

We summarized continuous variables as mean ± standard deviation, and categorical variables as counts and percentages. We compared groups using Student’s *t*-test for continuous variables and the χ^2^ test for categorical variables.

We examined fatigue and HRQoL separately according to DAS28- and RAPID3-defined remission and LDA. We initially stratified descriptive comparisons with controls by sex.

To assess the potential impact of missing RAPID3 data, participants with and without available RAPID3 assessments were compared with respect to age, BMI, FACIT-F, SF-12, DAS28 (continuous score and disease activity category), and hemoglobin levels.

To assess potential effect modification by sex, an initial general linear model including a sex-by-disease-activity-state interaction term was fitted for each comparison. As no statistically significant interactions were observed, the final analyses were performed using multivariable linear regression models. Separate models were fitted for each comparison (controls vs. DAS28 remission, controls vs. DAS28 LDA, controls vs. RAPID3 remission, and controls vs. RAPID3 LDA), adjusting for age, sex, BMI, and hemoglobin concentration. These covariates were selected because of their clinical relevance and their consistent availability in both the RA and control groups, allowing the same adjustment strategy to be applied across all comparisons. Regression coefficients (β), 95% confidence intervals (CIs), and two-sided *p*-values were reported. The three patient-reported outcomes (FACIT-F, SF-12 PCS, and SF-12 MCS) were used to address the primary research question. The 12 comparisons between DAS28- or RAPID3-defined remission or low disease activity and controls, across the three patient-reported outcomes, were considered the principal outcome comparisons. To account for multiple comparisons, Holm’s step-down procedure was applied across these 12 comparisons, and Holm-adjusted *p*-values were reported. Model assumptions were evaluated by inspection of residual plots, and no relevant deviations from linear regression assumptions were identified. Analyses were performed using complete-case data, and no imputation of missing values was undertaken.

We evaluated agreement between DAS28 and RAPID3 disease activity categories using weighted Cohen’s kappa coefficients with linear and quadratic weights. We considered quadratic weighting the primary measure because of the ordinal nature of the categories and because it assigns progressively greater weight to larger discrepancies between categories. We also quantified exact agreement and the proportions of classifications differing by one, two, or three disease activity categories. Agreement was evaluated overall, according to sex and age (<65 versus ≥65 years), and across combined age-by-sex subgroups. Quadratic weighted kappa coefficients and their 95% confidence intervals were estimated using bootstrap resampling, which was also used to compare kappa coefficients between sexes. To determine whether the relationship between both classifications differed by sex, we fitted an ordinal logistic regression model including DAS28 category, sex, age, and the DAS28-by-sex interaction. Marginal symmetry between DAS28 and RAPID3 classifications was assessed using the Bowker test of symmetry. Finally, we classified RAPID3 as lower than, concordant with, or higher than DAS28 and compared the distribution of these categories across age- and sex-defined groups using the χ^2^ test. These analyses were considered secondary or exploratory.

We performed all analyses using IBM SPSS Statistics version 29.0 (IBM Corp., Armonk, NY, USA). Bootstrap estimates were based on 10,000 resamples. All tests were two-sided, with *p* < 0.05 considered statistically significant.

### 2.8. Ethical Considerations

This study was initially approved by the Research Ethics Committee of Bellvitge University Hospital on 12 March 2020 (reference PR057/20). A subsequent amendment to the same study protocol was approved on 13 October 2022. This study was conducted in accordance with the principles of the Declaration of Helsinki, and written informed consent was obtained from all participants before enrolment.

## 3. Results

### 3.1. Study Population

**Figure 1:**
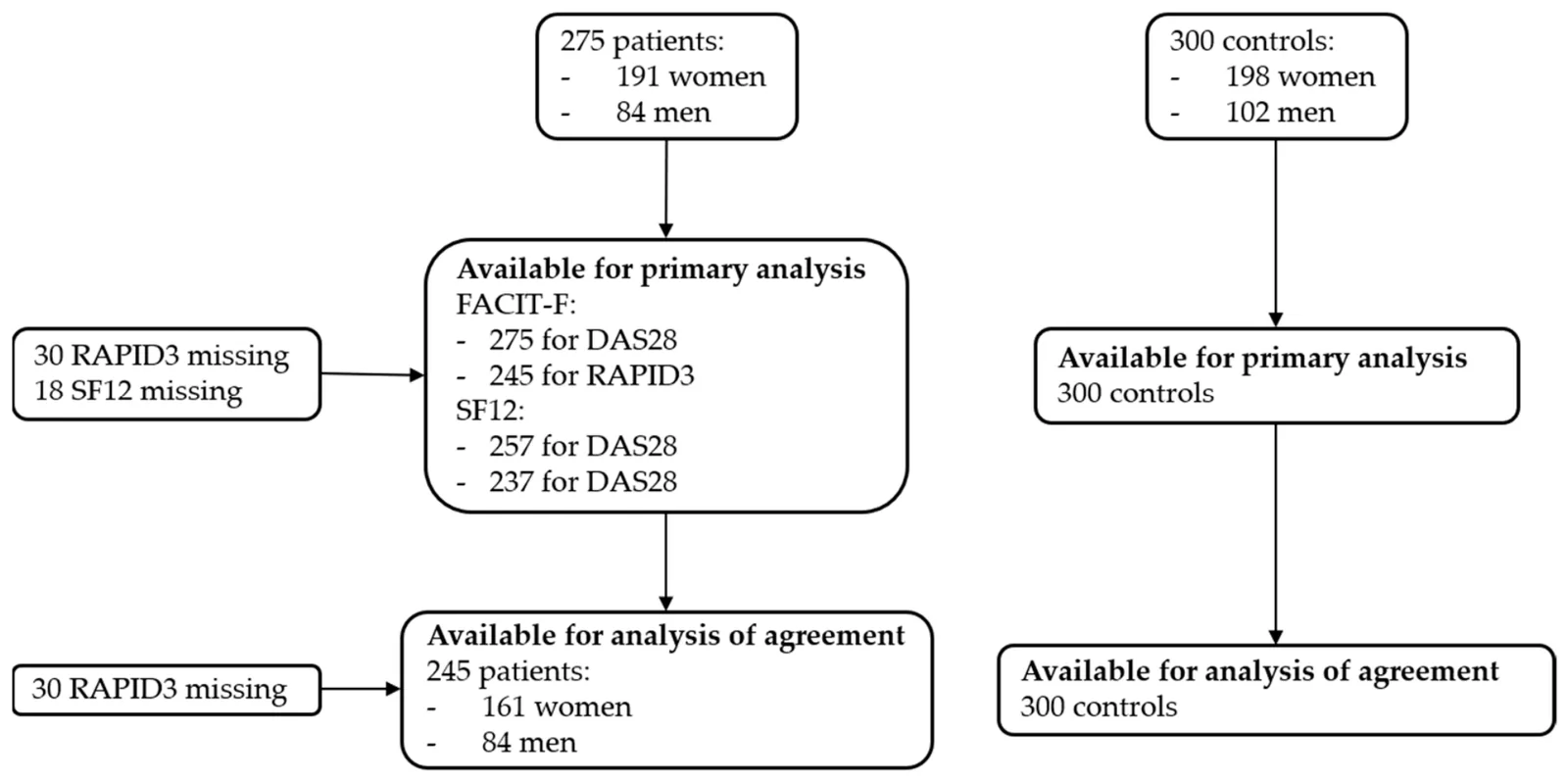
Participant flow diagram.

A total of 275 patients with RA and 300 controls were included. The flow of participants through the different analyses is shown in Figure 1.

Baseline demographic and clinical characteristics of the RA cohort, overall and stratified by sex, are shown in Table 1. Patients with available RAPID3 data (n = 245) and those with missing RAPID3 data (n = 30) were similar in age, BMI, FACIT-F scores, SF-12 physical and mental component scores, and DAS28 disease activity category distributions. However, patients with missing RAPID3 data had slightly higher DAS28 scores (median 3.01 vs. 2.67; *p* = 0.046) and lower hemoglobin levels (median 13.2 vs. 13.8 g/dL; *p* = 0.039). All participants with missing RAPID3 data were women.

### 3.2. Fatigue and Health-Related Quality of Life According to Disease Activity State

Fatigue and HRQoL according to DAS28- and RAPID3-defined disease activity states are shown in Table 2.

Among women, mean FACIT-F scores were 40.2 ± 8.2 in DAS28 remission, 35.9 ± 9.9 in DAS28 LDA, 43.4 ± 6.9 in RAPID3 remission and 41.2 ± 5.2 in RAPID3 LDA, compared with 40.1 ± 10.0 in controls. FACIT-F scores were significantly lower in women with DAS28-defined low disease activity than in controls (*p* < 0.01). The proportion of women with FACIT-F < 40 was 49%, 60%, 29% and 39%, respectively, compared with 37% among controls, and was significantly higher in the DAS28 LDA group (*p* < 0.01).

SF-12 physical component scores in women were 39.9 ± 9.5 in DAS28 remission, 39.0 ± 8.6 in DAS28 LDA, 44.4 ± 8.8 in RAPID3 remission and 40.1 ± 8.5 in RAPID3 LDA, compared with 44.0 ± 11.5 in controls. Physical component scores were significantly lower in women in DAS28 remission and DAS28 LDA than in controls (both *p* < 0.01). Corresponding SF-12 mental component scores were 48.9 ± 10.3, 42.8 ± 12.0, 49.2 ± 11.1 and 46.1 ± 10.1, respectively, compared with 50.2 ± 10.2 in controls. Mental component scores were significantly lower in women with DAS28-defined LDA (*p* < 0.001).

Among men, mean FACIT-F scores were 43.0 ± 6.1 in DAS28 remission, 42.3 ± 6.6 in DAS28 LDA, 44.3 ± 5.1 in RAPID3 remission and 43.8 ± 5.5 in RAPID3 LDA, compared with 42.5 ± 9.3 in controls. The proportion with FACIT-F < 40 was 21%, 36%, 19% and 27%, respectively, compared with 29% among controls. SF-12 physical component scores were 44.5 ± 9.0, 50.9 ± 9.9, 47.1 ± 7.7 and 43.0 ± 10.2, compared with 46.7 ± 10.6 in controls, while corresponding mental component scores were 52.5 ± 9.6, 49.8 ± 11.1, 54.8 ± 8.2 and 49.8 ± 10.6, respectively, compared with 50.9 ± 9.9 in controls. No statistically significant differences were observed among men.

### 3.3. Adjusted Differences Compared with Controls

**Figure 2:**
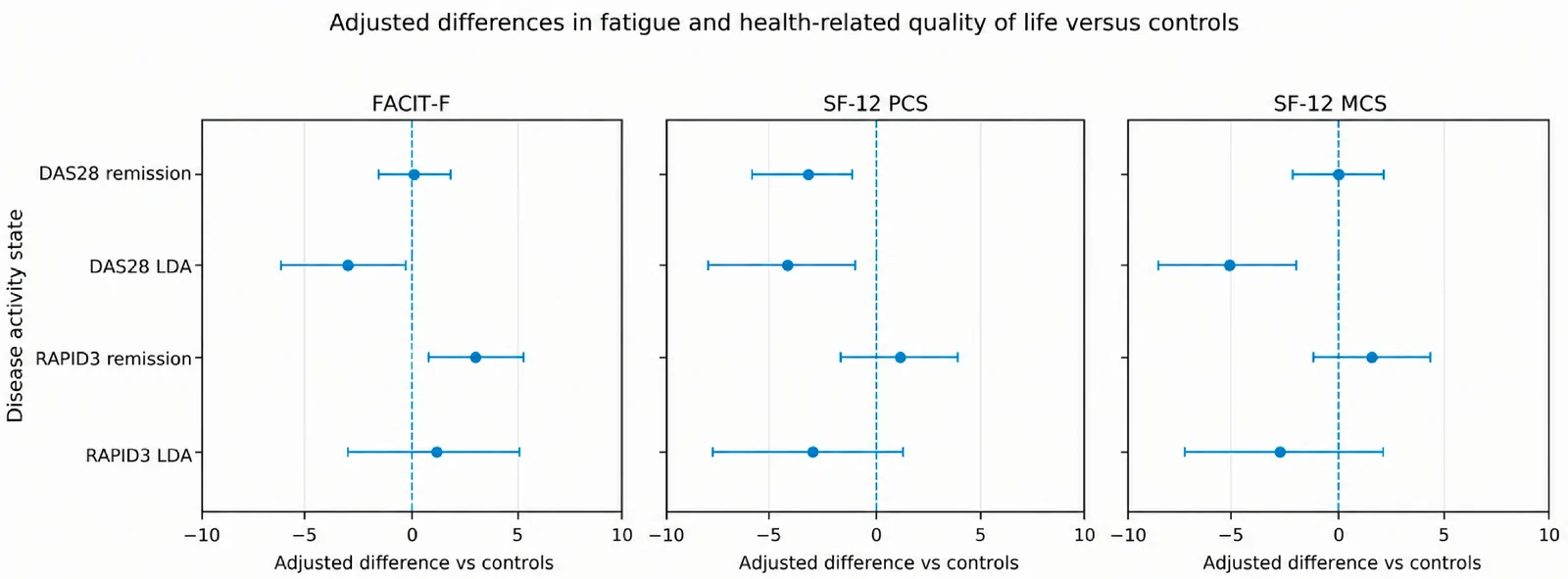
Adjusted differences in fatigue and health-related quality of life between disease activity states and controls. Point estimates represent adjusted mean differences and horizontal bars indicate 95% confidence intervals. The vertical reference line at zero indicates no difference from controls.

We used multivariable linear regression models adjusted for age, sex, BMI, and hemoglobin to estimate differences in fatigue and HRQoL between each disease activity state and controls. Table 3 presents the adjusted estimates, and Figure 2 displays them graphically.

Among patients in DAS28 remission, no statistically significant difference from controls was detected for FACIT-F (adjusted difference 0.12, 95% CI −1.77 to 2.02; *p* = 0.897) or SF-12 MCS (−0.07, 95% CI −2.23 to 2.10; *p* = 0.952). However, SF-12 PCS remained significantly lower than in controls (adjusted difference −3.37, 95% CI −5.56 to −1.18; nominal *p* = 0.003; Holm-adjusted *p* = 0.033).

Patients with DAS28-defined LDA had lower FACIT-F scores than controls (adjusted difference −2.90, 95% CI −5.55 to −0.25; nominal *p* = 0.032), although this difference did not remain statistically significant after Holm adjustment (adjusted *p* = 0.256). SF-12 PCS scores were also lower (adjusted difference −3.91, 95% CI −6.89 to −0.93; nominal *p* = 0.010), but this association did not remain statistically significant after adjustment (adjusted *p* = 0.100). In contrast, SF-12 MCS scores remained significantly lower after Holm adjustment (adjusted difference −5.15, 95% CI −8.14 to −2.15; nominal *p* = 0.001; adjusted *p* = 0.012). Patients in RAPID3 remission had higher FACIT-F scores than controls (2.94, 95% CI 0.62 to 5.25; nominal *p* = 0.013). However, this difference did not remain statistically significant after adjustment for multiple comparisons (Holm-adjusted *p* = 0.117); SF-12 physical (1.06, 95% CI −1.56 to 3.69; *p* = 0.426) and SF12 MCS (1.48, 95% CI −1.12 to 4.08; *p* = 0.264) did not differ significantly from controls.

No significant differences from controls were observed among patients with RAPID3-defined LDA for FACIT-F (1.04, 95% CI −2.90 to 4.96; *p* = 0.602), SF-12 PCS (−3.62, 95% CI −8.17 to 0.93; *p* = 0.119) or SF-12 MCS (−2.73, 95% CI −7.17 to 1.70; *p* = 0.226).

### 3.4. Agreement Between DAS28 and RAPID3 Disease Activity Classifications

Overall exact agreement between DAS28 and RAPID3 was 36.7%. A difference of one category was observed in 34.3% of participants, a difference of two categories in 23.7%, and a difference of three categories in 5.3%. Linearly weighted κ = 0.25 (95% CI 0.18–0.33), while quadratically weighted κ = 0.35 (95% CI 0.26–0.44). Quadratic weighted agreement was κ = 0.30 (95% CI 0.19–0.40) in women and κ = 0.41 (95% CI 0.20–0.58) in men. Although agreement was numerically higher in men, the difference between sexes was not statistically significant (Δκ = 0.11; 95% CI −0.12 to 0.31; bootstrap *p* = 0.34). Consistently, ordinal logistic regression showed no significant interaction between DAS28 category and sex in predicting RAPID3 disease activity category (interaction *p* = 0.968).

Agreement tended to be higher in patients aged < 65 years than in those aged ≥ 65 years (quadratic-weighted κ = 0.40 vs. 0.32). However, neither the age-specific differences nor those observed in the combined age- and sex-stratified analyses reached statistical significance.

**Figure 3:**
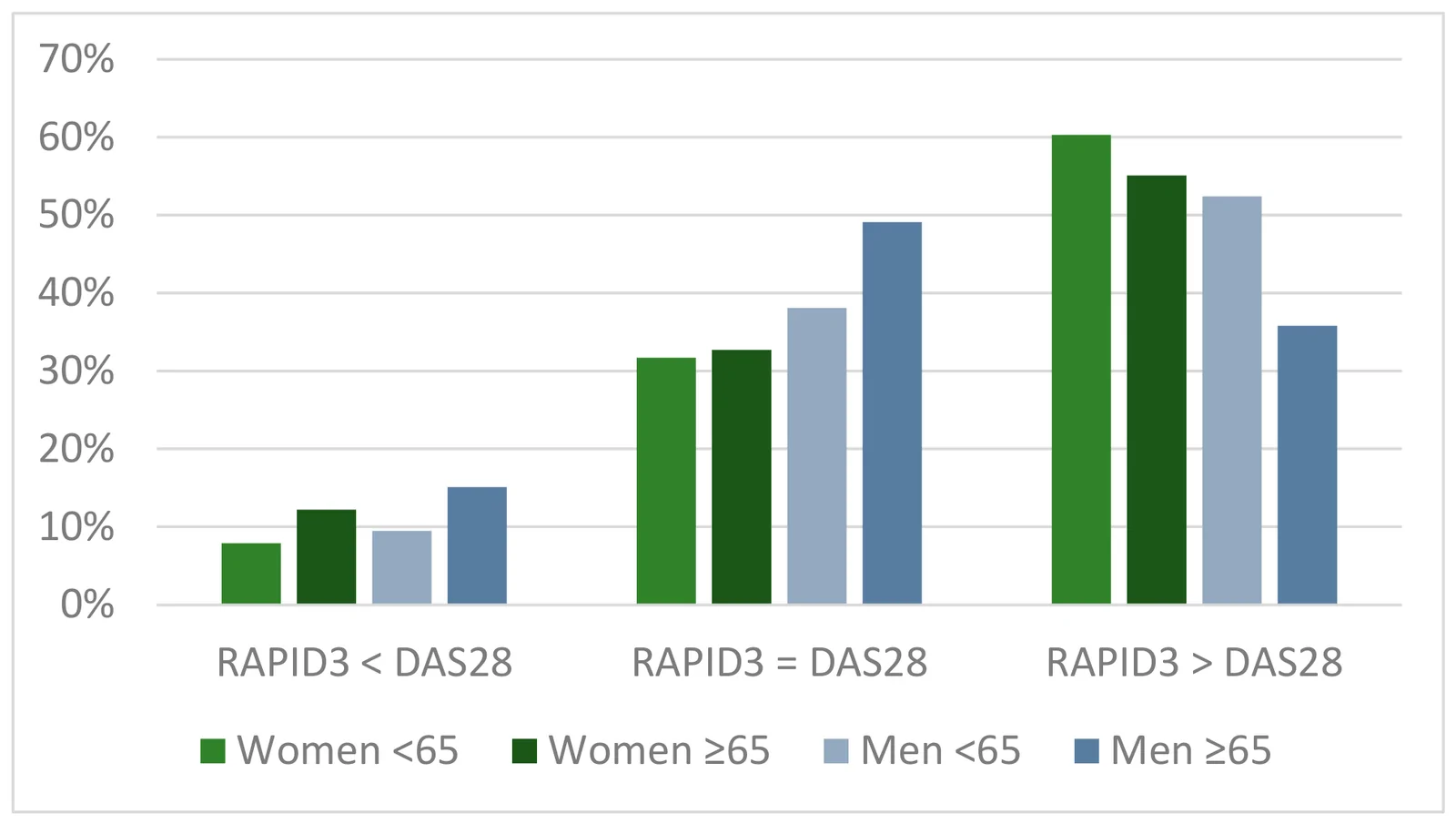
Concordance and direction of disagreement between DAS28 and RAPID3 according to age and sex. Data are presented as percentages of patients within each subgroup.

RAPID3 classified patients into higher disease activity categories than DAS28 in 125 of 155 discordant classifications (80.6%), whereas it classified patients into lower categories in 30 (19.4%). The Bowker test of symmetry demonstrated significant marginal asymmetry between DAS28 and RAPID3 classifications (χ^2^ = 80.03, df = 6, *p* < 0.001), supporting a directional pattern of discordance towards higher RAPID3 disease activity categories (Figure 3). Despite this overall asymmetry, the distribution of lower, concordant, and higher RAPID3 classifications relative to DAS28 did not differ significantly according to age and sex (χ^2^ = 9.24, df = 6, *p* = 0.160).

The full 4 × 4 cross-tabulation of DAS28 and RAPID3 disease activity categories is provided in Supplementary Table S1.

## 4. Discussion

In this study, we examined differences in fatigue and HRQoL between patients with RA classified as being in remission or LDA and individuals without inflammatory arthritis, using DAS28- and RAPID3-defined disease activity states separately. Three main findings emerged. First, patients in DAS28 remission had no statistically significant differences from controls in fatigue or mental HRQoL, although physical HRQoL remained impaired, whereas DAS28-defined LDA was associated with poorer mental HRQoL. Second, no statistically significant differences from controls were observed in either RAPID3-defined remission or LDA. Third, agreement between DAS28 and RAPID3 classifications was limited and directional, with RAPID3 more frequently assigning patients to higher disease activity categories. Overall, these findings indicate that residual impairment in patients with RA may persist even when DAS28-defined remission or LDA is achieved, particularly in specific dimensions of health.

The different patterns observed for DAS28-defined remission and LDA suggest that achieving remission may be associated with a more favorable health status than remaining in LDA, although residual impairment may persist even among patients classified as being in remission. In our study, physical HRQoL remained significantly lower than in controls among patients in DAS28 remission, whereas mental HRQoL was significantly lower among those in DAS28-defined LDA. This pattern is consistent with previous evidence showing that patients who achieve sustained remission experience better fatigue, function, and physical and mental HRQoL than those who remain in sustained LDA [6]. Residual pain and fatigue may nevertheless persist among patients fulfilling clinical remission criteria and may not necessarily reflect ongoing synovial inflammation [3,16,23]. We believe that the residual physical impairment observed in remission may reflect cumulative structural damage, longstanding disability, musculoskeletal comorbidity, or age-related loss of function rather than ongoing inflammatory activity, and should not by itself be interpreted as an indication for greater immunomodulation.

Although both remission and LDA are accepted treatment targets within treat-to-target strategies, our findings suggest that they should not be regarded as interchangeable from the perspective of fatigue and HRQoL. Patients with DAS28-defined LDA remained distinguishable from controls across all three outcomes, indicating a broader residual burden than that observed in remission. This interpretation is supported by longitudinal evidence showing persistently poorer patient-reported outcomes in sustained LDA than in sustained remission [6], as well as by individual patient-data analyses indicating that remission and LDA may differ in their capacity to prevent radiographic progression [7]. We consider that this does not imply that LDA is an inadequate target when remission is not achievable, but it does qualify its clinical meaning beyond the numerical range of the composite score.

A different pattern emerged when disease activity was defined using RAPID3. Neither RAPID3-defined remission nor LDA was associated with statistically significant differences from controls in fatigue or HRQoL after adjustment for multiple comparisons. We interpret these findings cautiously: they do not indicate that RAPID3 is superior to DAS28 for assessing inflammatory disease activity. Because RAPID3 is based entirely on patient-reported physical function, pain, and global assessment [10], patients with low scores are partly selected on the basis of favorable perceived health. The absence of statistically significant differences in fatigue and HRQoL in RAPID3-defined remission should therefore be interpreted in light of this conceptual overlap and does not constitute evidence of normalization or of superior inflammatory control. Previous comparisons have similarly shown that RAPID3 and conventional composite indices are related but not interchangeable [11,12]. We believe that DAS28 and RAPID3 should therefore be regarded as complementary rather than competing measures: DAS28 incorporates joint examination and an inflammatory marker, whereas RAPID3 reflects the patient’s experience of symptoms, function, and overall disease impact.

The magnitude of the observed differences should also be considered alongside their statistical significance. For FACIT-F, a minimally important difference of approximately 3–4 points has been established in patients with RA [20]. Because neither of these FACIT-F differences remained statistically significant after adjustment for multiple comparisons, they should not be emphasized as primary findings. For SF-12 component scores, clinically meaningful thresholds are less firmly established in RA, and we therefore avoided applying a fixed cut-off [24]. The observed PCS and MCS differences should instead be interpreted in the context of their magnitude and confidence intervals. More generally, published minimally important differences are largely derived from longitudinal within-patient changes and should therefore be applied cautiously to cross-sectional between-group comparisons such as those reported here [20,24].

The agreement analysis supports this interpretation. Concordance between DAS28 and RAPID3 was limited, and discordance was directional, with RAPID3 more frequently assigning patients to higher disease activity categories. Previous studies have also reported incomplete agreement between RAPID3 and examination-based composite indices and have cautioned against using RAPID3 alone as a measure of inflammatory disease activity [11,12]. We believe that this pattern reflects the different composition of the instruments and the influence on RAPID3 of pain, functional limitation, osteoarthritis, fatigue, and comorbidity that may not correspond to active inflammation. Pain and tender joint burden have been identified as important determinants of patient–physician discordance in global disease activity assessment [25], and recent evidence further suggests that disproportionate subjective disease activity components may be associated with nociplastic pain mechanisms [26]. Although these factors may be particularly relevant in middle-aged and older patients, neither age nor sex significantly modified the relationship between the two classifications, suggesting that this directional discordance is not confined to a particular demographic subgroup.

In our opinion, these findings also have implications for the interpretation of treat-to-target strategies. Composite indices remain essential for therapeutic decision-making in RA, but disease control should not be considered synonymous with complete restoration of health. Previous systematic evidence has emphasized that reliance exclusively on conventional disease activity targets may underestimate residual pain, fatigue, functional impairment, and compromised quality of life [3]. Real-world evidence also indicates that, although remission remains the predominant therapeutic goal, symptom control and reduction in the impact of RA on quality of life are also important treatment priorities [27]. Our results suggest that assessment of patients who have achieved DAS28 remission or LDA may benefit from a complementary evaluation of fatigue and HRQoL. Persistent patient-reported burden in this setting should prompt consideration of its underlying determinants. A patient-centered instrument such as RAPID3 may also help determine the extent to which disease control is accompanied by improvement in the dimensions of health most directly experienced by patients.

Several limitations should be acknowledged. The cross-sectional design precludes causal inference and does not establish whether movement from LDA to remission is followed by improvement in fatigue or HRQoL within individual patients. In addition, participants were recruited from a single tertiary center and were all older than 50 years, which may limit generalizability to other healthcare settings and younger RA populations. Although the adjusted models included age, sex, BMI, and hemoglobin, residual confounding remains possible because relevant determinants of fatigue and HRQoL, including multimorbidity, osteoarthritis, chronic pain, sleep disturbance, psychological factors, physical activity, socioeconomic status, and structural joint damage, were not incorporated. Several of these variables, as well as medications potentially associated with fatigue, were not systematically available in the control group and therefore could not be consistently included in the adjusted analyses. In addition, some symptom- and function-related variables may partly lie on the causal pathway between disease status and the patient-reported outcomes evaluated, making their inclusion as conventional confounders potentially problematic. Moreover, although controls were recruited from the same healthcare environment and were subject to similar exclusion criteria, unmeasured differences between groups cannot be excluded. Controls recruited because of musculoskeletal complaints had non-inflammatory, predominantly soft-tissue conditions without marked functional limitation, and individuals with markedly disabling musculoskeletal disease were not included. Nevertheless, even minor musculoskeletal symptoms may influence fatigue or physical HRQoL and could therefore have attenuated some between-group differences. Conversely, the exclusion of individuals with major systemic disease may have resulted in a control group with a lower burden of major systemic disease.

The unequal distribution of participants across disease activity categories reduced precision in some analyses, particularly for RAPID3-defined LDA, and multiple comparisons increased the possibility of type I error, which was specifically addressed using Holm adjustment. Some conceptual overlap also exists between RAPID3 and the outcomes evaluated, because all capture aspects of patient-perceived function and health; therefore, associations between RAPID3 and patient-reported outcomes should be interpreted cautiously because of these shared constructs. Furthermore, remission was defined only using DAS28 and RAPID3, without examining Boolean, SDAI, or CDAI criteria, and imaging was not available to assess possible subclinical synovitis. This is relevant because ultrasound-detected synovitis may persist in a substantial proportion of patients with longstanding clinical remission [28]. Finally, DAS28- and RAPID3-defined states were compared separately with controls for fatigue and HRQoL, while agreement between the two classifications was assessed directly. However, this study was not designed to determine whether one instrument was superior to the other in identifying patients with better or worse patient-reported health.

An important consideration when interpreting these findings is that the absence of a statistically significant difference from controls does not constitute formal evidence of equivalence. This study was not designed as an equivalence or non-inferiority analysis; no equivalence margins were prespecified, and some RAPID3 subgroups, particularly LDA, were small. Accordingly, non-significant findings should be interpreted as an absence of statistically detected differences rather than evidence of equivalent health status.

Despite these limitations, this study has several strengths. It was based on a relatively large, real-world cohort of 275 patients with RA and 300 controls recruited within the same healthcare environment. The inclusion of individuals without inflammatory arthritis provided an external benchmark against which the residual impact associated with remission and LDA could be interpreted. Comparisons restricted to disease activity categories within RA can demonstrate relative differences but cannot establish how closely a treatment target approaches the health status observed outside the disease. The simultaneous availability of DAS28 and RAPID3 also allowed disease control to be examined from both a conventional composite perspective and a fully patient-reported perspective. Fatigue and HRQoL were assessed using established instruments in both patients and controls, and the analyses were adjusted for relevant covariates. Agreement was evaluated using weighted kappa coefficients with bootstrap confidence intervals and complemented by an assessment of the direction of discordance.

## 5. Conclusions

DAS28-defined remission and LDA were associated with different patterns of residual impairment compared with controls. Patients in DAS28 remission had significantly lower physical HRQoL, whereas those in DAS28-defined LDA had significantly poorer mental HRQoL. No statistically significant differences in fatigue were observed for DAS28-defined remission or LDA, while neither RAPID3-defined remission nor LDA was associated with statistically significant differences in fatigue or HRQoL after adjustment for multiple comparisons. These findings do not indicate that either disease activity state represents complete normalization of health, nor do they support superiority of one instrument over the other.

The limited and directional agreement between DAS28 and RAPID3 indicates that the two instruments capture partly distinct dimensions of RA status. DAS28 provides a composite assessment incorporating clinical joint findings and an inflammatory marker, whereas RAPID3 predominantly reflects the patient-perceived burden of symptoms, function, and overall disease impact. We believe that inflammatory control and patient-reported health should therefore be regarded as complementary components of disease assessment. Prospective studies are needed to determine whether fatigue and HRQoL improve over time after patients achieve and sustain different disease activity states.

## Figures and Tables

**Table 1:** Demographic and clinical characteristics of patients with rheumatoid arthritis.

	All n: 275	Men n: 84	Women n: 191	*p*-Value
Age (years)	68.8 ± 9.0	71.9 ± 8.5	67.5 ± 8.8	<0.001
BMI (kg/m^2^)	27.8 ± 4.9	27.5 ± 3.5	27.9 ± 5.4	ns
Underweight (n, %)	1 (0.4%)	1 (1.2%)	0	ns
Normal range (n, %)	82 (29.8%)	18 (21.4%)	64 (33.5%)	-
Overweight (n, %)	121 (44.0%)	49 (58.3%)	72 (37.5%)	<0.01
Obese (n, %)	71 (25.8%)	16 (19.1%)	55 (29%)	-
Smoking				ns
Never (n, %)	239 (86.9%)	72 (85.7%)	164 (87.2%)	
Former (n, %)	3 (1.1%)	1 (1.2%)	2 (1.1%)	
Current (n, %)	33 (12.0%)	11 (13.1%)	22 (11.7%)	
Physical activity, n: 271				ns
None (n, %)	125 (46.1%)	31 (37.0%)	94 (50.3%)	
Sporadic (n, %)	52 (19.2%)	16 (19.0%)	36 (19.3%)	
Regular with low intensity (n, %)	88 (32.5%)	33 (39.2%)	55 (29.4%)	
Regular with high intensity (n, %)	6 (2.2%)	4 (4.8%)	2 (1.0%)	
Hemoglobin (g/dL)	13.6 ± 1.3	14.2 ± 1.5	13.3 ± 1.2	<0.001
Serum albumin (g/L)	43.8 ± 3.7	42.9 ± 4.7	44.3 ± 3.5	<0.01
Disease duration (years), n: 267	15.5 ± 10.3	12.5 ± 9.6	16.8 ± 10.3	<0.01
RF-positive (n, %), n: 248	173 (68.9%)	50/83 (60.2%)	123/168 (73.0%)	ns
RF titer (IU/L)	177 ± 399	175 ± 224	208 ± 415	ns
ACPA-positive (n, %), n: 249	167 (66.8%)	52/83 (62.6%)	115/167 (69%)	ns
ACPA titer (U/L)	400 ± 711	571 ± 1040	369 ± 672	ns
Current medication				
Glucocorticoids (n, %)	135 (49.1%)	46 (54.7%)	89 (46.5%)	ns
csDMARDs (n, %)	245 (89.1%)	73 (86.9%)	172 (90.0%)	ns
bDMARDs (n, %)	88 (32.0%)	20 (23.8%)	68 (36.0%)	<0.001
JAK inhibitors (n, %)	12 (4.4%)	2 (2.4%)	10 (5.0%)	ns
ESR (mm/h)	24.67 ± 23.6	24.3 ± 26.2	24.8 ± 20.8	ns
CRP (mg/L)	7.2 ± 12.7	10.1 ± 18.3	5.2 ± 6.1	<0.05
DAS28	2.8 ± 1.1	2.5 ± 1.2	2.9 ± 1.1	<0.01
Remission (n, %)	126 (45.9%)	49 (58.3%)	77 (40.5%)	ns
LDA (n, %)	63 (22.9%)	16 (19%)	47 (24.5%)	
MDA (n, %)	76 (27.6%)	15 (17.9%)	61 (32%)	
HDA (n, %)	10 (3.6%)	4 (4.8%)	6 (3%)	
RAPID3, n: 245	8.6 ± 6.7	37 (44.0%)	9.7 ± 6.9	<0.001
Remission (n, %)	75 (30.6%)	11 (13.1%)	38 (24%)	<0.01
LDA (n, %)	24 (9.8%)	27 (32.2%)	13 (8%)	
MDA (n, %)	82 (33.5%)	9 (10.7%)	55 (34%)	
HDA (n, %)	64 (26.1%)		55 (34%)	
FACIT-F	37.7 ± 10.0	41.6 ± 6.5	36.0 ± 10.6	<0.001
FACIT-F < 40, n (%)	143 (52.0%)	27 (32.1%)	116 (60.7%)	<0.001
SF-12, n: 257				
MCS	47.1 ± 11.3	51.5 ± 10.0	45.1 ± 11.4	<0.001
PCS	38.6 ± 9.9	42.5 ± 9.6	36.8 ± 9.5	<0.001

Data were not available for all variables. The number of participants with available data (n) is indicated for variables with missing data. Percentages were calculated using the number of participants with available data as the denominator. BMI, body mass index; RF, rheumatoid factor; ACPA, anti-citrullinated protein antibodies; ESR, erythrocyte sedimentation rate; CRP, C-reactive protein; LDA, low disease activity; MDA, moderate disease activity; HDA, high disease activity. SF-12 MCS, 12-item Short Form Health Survey Mental Component Summary; SF-12 PCS, 12-item Short Form Health Survey Physical Component Summary. *p*-values correspond to comparisons between men and women.

**Table 2:** Fatigue and health-related quality of life according to DAS28- and RAPID3-defined disease activity states and controls.

	**DAS28 Remission**	**RAPID3 Remission**	**DAS28** **LDA**	**RAPID3** **LDA**	**Controls**
	**Men**				
	n: 49	n: 37	n: 16	n: 11	n: 102
Age, years	71.2 ± 8.6	72.8 ± 7.8	72.7 ± 9.5	67.1 ± 8.0	71.1 ± 9.2
BMI, kg/m^2^	27.6 ± 3.6	27.7 ± 3.0	27.4 ± 2.4	30.6 ± 3.3	27.4 ± 4.4
Hemoglobin, g/dL	14.5 ± 1.3	14.4 ± 1.3	13.9 ± 1.8	14.6 ± 1.2	14.5 ± 1.6
FACIT-F FACIT-F < 40, n (%)	43.0 ± 6.1 9 (18%)	44.3 ± 5.1 7 (19%)	42.3 ± 6.6 6 (38%)	43.8 ± 5.5 3 (27%)	42.5 ± 9.3 30 (29%)
SF-12					
MCS	52.5 ± 9.6	54.8 ± 8.2	49.8 ± 11.1	49.8 ± 10.6	50.9 ± 9.9
PCS	44.5 ± 9.0	47.1 ± 7.7	50.9 ± 9.9	43.0 ± 10.2	46.7 ± 10.6
	**Women**				
	n: 77	n: 38	n: 47	n: 13	n: 198
Age, years	66.6 ± 7.8	68.5 ± 8.7	68.1 ± 10.0	66.7 ± 6.1	67.3 ± 9.2
BMI, kg/m^2^	27.7 ± 5.4	26.9 ± 4.6	27.8 ± 5.3	28.4 ± 6.7	27.8 ± 5.3
Hemoglobin, g/dL	13.6 ± 1.1	13.3 ± 1.2	13.2 ± 1.1	13.2 ± 1.4	13.7 ± 1.1
FACIT-F	40.2 ± 8.2	43.4 ± 6.9	35.9 ± 9.9	41.2 ± 5.2	40.1 ± 10.0
FACIT-F < 40, n (%)	33 (43%)	11 (29%)	30 (64%)	5 (39%)	74 (37%)
SF-12					
MCS	48.9 ± 10.3	49.2 ± 11.1	42.8 ± 12.0	46.1 ± 10.1	50.2 ± 10.2
PCS	39.9 ± 9.5	44.4 ± 8.8	39.0 ± 8.6	40.1 ± 8.5	44.0 ± 11.5

BMI, body mass index; FACIT-F, Functional Assessment of Chronic Illness Therapy–Fatigue; SF-12 MCS, 12-item Short Form Health Survey Mental Component Summary; DAS28, Disease Activity Score in 28 joints; RAPID3, Routine Assessment of Patient Index Data 3; SF-12 PCS, 12-item Short Form Health Survey Physical Component Summary; LDA, low disease activity.

**Table 3:** Adjusted differences in fatigue and health-related quality of life between DAS28- and RAPID3-defined disease activity states and controls.

β (95% CI) n = Patients/Controls *p*-Value	Adjusted FACIT-F Difference vs. Controls	Adjusted SF-12 PCS Difference vs. Controls	Adjusted SF-12 MCS Difference vs. Controls
**DAS28 remission**	0.12 (−1.77 to 2.02)	−3.37 (−5.56 to −1.18)	−0.07 (−2.23 to 2.10)
	n = 126/273	n = 121/273	n = 121/273
	*p* = 0.897	*p* = 0.003	*p* = 0.952
**DAS28 LDA**	−2.90 (−5.55 to −0.25)	−3.91 (−6.89 to −0.93)	−5.15 (−8.14 to −2.15)
	n = 63/273	n = 58/273	n = 58/273
	*p* = 0.032	*p* = 0.010	*p* = 0.001
**RAPID3 remission**	2.94 (0.62 to 5.25)	1.06 (−1.56 to 3.69)	1.48 (−1.12 to 4.08)
	n = 75/273	n = 75/273	n = 75/273
	*p* = 0.013	*p* = 0.426	*p* = 0.264
**RAPID3 LDA**	1.04 (−2.90 to 4.96)	−3.62 (−8.17 to 0.93)	−2.73 (−7.17 to 1.70)
	n = 24/273	n = 22/273	n = 22/273
	*p* = 0.602	*p* = 0.119	*p* = 0.226

DAS28, Disease Activity Score in 28 joints; RAPID3, Routine Assessment of Patient Index Data 3; LDA, low disease activity; SF-12 PCS, 12-item Short Form Health Survey Physical Component Summary; SF-12 MCS, 12-item Short Form Health Survey Mental Component Summary.

## Data Availability

The datasets used and analyzed during the current study are available from the corresponding author upon reasonable request.

## Supplementary Materials

The following supporting information can be downloaded at: https://www.mdpi.com/article/10.3390/jcm15187133/s1, Supplementary Table S1: Full 4 × 4 cross-tabulation of DAS28 and RAPID3 disease activity categories.
