# Supplementary material for: Beyond Disease Activity: Fatigue and Health-Related Quality of Life in Rheumatoid Arthritis Remission and Low Disease Activity
Source: J Clin Med. 2026 Sep 14;15(18):7133. doi: 10.3390/jcm15187133 (PMC13607491; doi:10.3390/jcm15187133)
Supplement: Supplementary file 1 [file jcm-15-07133-s001.zip › jcm-4513311-supplementary.pdf]

**Supplementary Table S1.** Full 4 × 4 cross-tabulation of DAS28 and RAPID3 disease activity categories.

| DAS28 \ RAPID3 | Remission | Low activity | Moderate activity | High activity | Total |
|---|---|---|---|---|---|
| **Remission** | 53 | 13 | 36 | 13 | 115 |
| **Low activity** | 16 | 5 | 19 | 16 | 56 |
| **Moderate activity** | 6 | 6 | 25 | 28 | 65 |
| **High activity** | 0 | 0 | 2 | 7 | 9 |
| **Total** | 75 | 24 | 82 | 64 | 245 |
